# The Comparison of ChatGPT 3.5, Microsoft Bing, and Google Gemini for Diagnosing Cases of Neuro-Ophthalmology

**DOI:** 10.7759/cureus.58232

**Published:** 2024-04-14

**Authors:** Ruchi Shukla, Ashutosh K Mishra, Nilakshi Banerjee, Archana Verma

**Affiliations:** 1 Department of Ophthalmology, All India Institute of Medical Sciences, Raebareli, Raebareli, IND; 2 Department of Neurology, All India Institute of Medical Sciences, Raebareli, Raebareli, IND

**Keywords:** google gemini, microsoft bing, artificial intelligence, neuro-ophthalmology, chatgpt

## Abstract

Objective: We aim to compare the capabilities of ChatGPT 3.5, Microsoft Bing, and Google Gemini in handling neuro-ophthalmological case scenarios.

Methods: Ten randomly chosen neuro-ophthalmological cases from a publicly accessible database were used to test the accuracy and suitability of all three models, and the case details were followed by the following query: "What is the most probable diagnosis?"

Results: On the basis of the accuracy of diagnosis, all three chat boxes (ChatGPT 3.5, Microsoft Bing, and Google Gemini) gave the correct diagnosis in four (40%) out of 10 cases, whereas in terms of suitability, ChatGPT 3.5, Microsoft Bing, and Google Gemini gave six (60%), five (50%), and five (50%) out of 10 case scenarios, respectively.

Conclusion: ChatGPT 3.5 performs better than the other two when it comes to handling neuro-ophthalmological case difficulties. These results highlight the potential benefits of developing artificial intelligence (AI) models for improving medical education and ocular diagnostics.

## Introduction

The use of deep learning (DL) and artificial intelligence (AI) in medicine, especially ophthalmology, has advanced significantly since 2015 [1]. Recently, there has been an increase in the use of natural language processing (NLP) in ophthalmology, which involves using AI to understand and converse with human language [2].

The publication of massive DL models known as foundation models has drawn a lot of media attention to NLP in recent months [3]. A subfield of artificial intelligence called "generative AI" is concerned with using a massive amount of current data to create new, original content such as writing, images, or audio.

NLP advances have made chatbots a potentially useful tool in the healthcare industry. Recently, there has been interest in assessing large language models' (LLMs) capacity to comprehend and produce natural language in the medical field [4].

LLMs have advanced to provide responses that are getting closer to those of humans with the use of a self-supervised learning process and extensive textual data training [5]. Because clinical reasoning frequently takes years to perfect through training and practical experience, the medical domain might present a substantial obstacle for LLMs.

AI models can be very useful in the field of ophthalmology, addressing concerns about care that are unique to each patient, offering prompt explanations of pertinent standards, and encouraging dialogues about eye ailments, procedures, and therapies. Though AI models such as ChatGPT have proven successful in the general domains of law, business, and medicine, they have been demonstrated to have inconsistent accuracy when responding to questions on specific medical specialties [6].

Online health information searches are becoming more common. A survey conducted in the United States found that about two out of every three people look up health information online, and one out of every three adults uses search engines to self-diagnose [7].

A vast range of web content is used in a self-supervised manner to train LLMs such as ChatGPT [8]. Even if there is a large amount of training data available on the internet, the quality of the material varies. This is especially troubling because LLMs are unable to assess the validity or consistency of the training data [9]. Furthermore, LLMs may not have domain-specific knowledge, which leaves them open to producing plausible but possibly false answers. Even with the quick development of LLMs, a more in-depth analysis of their performance in particular medical fields is still necessary.

Specialization in neuro-ophthalmology addresses neurological issues pertaining to the eye. This specialized field primarily studies problems that impact the eye's motions, visual pathways, and visual processing. In order to diagnose disorders that can potentially be life-threatening or vision-threatening, a comprehensive history, examination, neuroimaging, and laboratory test are all necessary in the intellectually taxing discipline of neuro-ophthalmology.

There have been some recent investigations into how well LLMs perform in the field of ophthalmology. An encouraging result of about 40%-50% was reported by Antaki et al. [10] and Mihalache et al. [11] in their evaluations of ChatGPT 3.5's performance on Ophthalmic Knowledge Assessment Program (OKAP) assessment questions. According to both authors, ChatGPT 3.5 performed worse on ophthalmology subspecialty questions than on general questions.

Most research has concentrated on the application of ChatGPT in the ophthalmology domain. There is still a need for a thorough assessment of Google Gemini and Microsoft Bing's diagnostic capacities for neuro-ophthalmological cases.

There has not been enough research done on the effectiveness and dependability of AI in the subspecialty of neuro-ophthalmology question answering. By comparing the various AI models' output to in-depth case descriptions of different neuro-ophthalmic diseases, we hope to learn more about the various AI models' capabilities and limits. In this study, we sought to assess and compare the performance of three freely available LLMs in answering questions for diagnosing neuro-ophthalmological cases: OpenAI's ChatGPT 3.5, Microsoft Bing, and Google Gemini. Our cases are derived from the neuro-ophthalmology subspecialty taken from "Neuro-Ophthalmology 2023: When Should I Worry? Concerning Signs, Symptoms, and Findings in Neuro-Ophthalmology," which is available online [12].

Our research may shed important light on the advantages and disadvantages of employing LLM chatbots to get answers in the subspeciality of neuro-ophthalmology.

## Materials and methods

For the purpose of the study, we utilized cases from the publicly accessible database of "Neuro-Ophthalmology 2023: When Should I Worry? Concerning Signs, Symptoms, and Findings in Neuro-Ophthalmology." We selected 10 case presentations with various neuro-ophthalmic diseases, including "ethambutol optic neuropathy," "optic neuritis in the setting of myelin oligodendrocyte glycoprotein (MOG) antibody−associated disease (MOGAD)," "sixth nerve palsy secondary to immunoglobulin G4 (IgG4)-related disease," "superior optic disc hypoplasia," "arteritic anterior ischemia on due to (d/t) giant cell arteritis," "pseudotumor cerebri," "trochlear nerve palsy," "vitreomacular traction," “amiodarone-associated toxic optic neuropathy," and "idiopathic orbital inflammatory syndrome."

Ten cases with confirmed diagnoses were randomly chosen. Each case was described with details about the patient's demographics, history, chief complaint, any pertinent ocular or medical histories, ophthalmic examination findings, and ocular imaging findings (when necessary). Table 1 displays the 10 case descriptions that were input into each of the three artificial intelligence systems.

**Table 1:** Displaying the 10 case descriptions that were input into each of the three artificial intelligence systems BCVA, best-corrected visual acuity; IOP, intraocular pressure; SITA, Swedish interactive thresholding algorithm; OU, oculus uterque; PICC, peripherally inserted central catheter; OCT, optical coherence tomography; PCP, primary care physician

Case Description	Diagnosis
1. A 32-year-old male (he/him) presented for progressive blurring of his vision. The patient reported that six weeks prior, he began noticing difficulty reading novels; this progressed to difficulty seeing his phone clearly and driving. He reported that the decline in his vision was bilateral and painless and occurred insidiously. He denied having flashes, floaters, complete loss of vision, or double vision. He denied headaches, weakness, numbness, and difficulties with coordination and walking. The patient was previously healthy but had been diagnosed with tuberculosis seven months prior, when he presented to the emergency department with fevers, chills, weight loss, and an intractable cough. He had emigrated from Pakistan one year prior. The patient was placed on rifampin, isoniazid, pyridoxine, and ethambutol (25 mg/kg/day). He did not consume alcohol, cigarettes, marijuana, or other substances, and he lived alone. On examination, this patient's visual acuity was 20/400 in the right eye (OD) and 20/300 in the left eye (OS). There was dyschromatopsia bilaterally, with 3/8 Ishihara plate correct OD and only the test plate correct OS. The pupils were equal, round, and reactive to light, without a relative afferent pupillary defect. Slit lamp and dilated fundus examinations were unremarkable, including normal optic nerves, maculae, vessels, and periphery. Humphrey visual field testing demonstrated cecocentral scotomas bilaterally. OCT showed normal retinal nerve fiber layer bilaterally compared to age-matched controls.	Ethambutol optic neuropathy
2. A 65-year-old male developed double vision, which occurred one week prior to presentation. Four days later, he developed blurred vision in the left eye, and the next morning, he woke up with complete vision loss of the left eye, which had not improved at the time of presentation. Vision in the right eye remained at baseline. He reported feeling well. However, on direct questioning, he reported mild fatigue, scalp tenderness, and right arm weakness for the last month. Two weeks prior, he noted that he had to take breaks while eating due to (d/t) jaw pain. He denied snoring but had never been evaluated for sleep apnea. His past medical history was notable for well-controlled hypertension, hyperlipidemia, and hypothyroidism, for which he was on stable doses of medications. He was a nonsmoker and drank wine occasionally. The ocular history was significant for early-stage primary open-angle glaucoma (on latanoprost {Xalatan}), and he had a normal eye examination a month prior. On examination at the urgent visit, the BCVA was 20/20 OD and light perception OS, with loss of color vision OS and the presence of a left afferent pupillary defect. Visual fields to confrontation were full OD and completely depressed OS. The IOP was 11 OU. He was pseudophakic in both eyes, and the posterior segment evaluation showed a normal right optic nerve, with cup-to-disc ratio of 0.45. The left optic nerve was pale and swollen, with a peripapillary cotton wool spot. The retinal vessels and peripheries were normal OU. Humphrey 24-2 SITA Standard visual field was normal in the right eye.	Arteritic anterior ischemic optic neuropathy due to giant cell arteritis
3. A 35-year-old Black female presented with right eye pain and vision loss. Ten days prior to presentation, she noted throbbing right eye pain that progressed in intensity over several days and became worse with eye movements. Two days prior to evaluation, she awoke with dim vision centrally in the right eye, "like a smudge," and presented to an optometrist, where visual acuity was 20/40 OD with noted red desaturation and a right relative afferent pupillary defect (RAPD) but intact visual field to confrontation. Fundus examination revealed right optic disc swelling. Her past medical history was notable for well-controlled hypertension, uterine fibroids, and benign glomus tympanicum tumor that was fully resected. She worked from home in human resources and did not use tobacco or drugs or drink alcohol. Family history was noncontributory. A month prior to the onset of symptoms, she had received the Pfizer COVID-19 booster shot and an influenza vaccine. She denied any sick contacts, animal exposures, or recent travel. She denied a history of headache, prior episodes of vision loss, pain on eye movements, diplopia, vertigo, dysarthria, dysphagia, weakness, numbness, paresthesias, difficulty with balance or walking, or bowel/bladder disturbances. On examination one day after the optometry visit, visual acuity was 20/200 in the right eye, with 1.5/11 Ishihara color plates and right RAPD. Humphrey 24-2 SITA Fast noted diffuse suppression OD and normal OS. Slit lamp examination was notable for normal anterior chamber examination, without cell or flare. Dilated fundus examination revealed a 270-degree right optic disc swelling without disc hemorrhage or pallor. The afferent and efferent examination of the left eye was normal.	Right optic neuritis in the setting of myelin oligodendrocyte glycoprotein (MOG) antibody-associated disease (MOGAD)
4. A 61-year-old male presented for the evaluation of new-onset horizontal double vision beginning three days prior to presentation. He reported that the double vision worsened when looking to his right. He denied any preceding injury, illness, or associated symptoms, such as numbness or weakness. However, he did note intermittent right-sided tinnitus for one month and mild swelling and tenderness behind his right ear beginning two days prior to presentation. His past medical history was significant for systemic hypertension, hyperlipidemia, and type 2 diabetes. His daily medications included atorvastatin 40 mg, hydrochlorothiazide 12.5 mg, and lisinopril 40 mg. His diabetes was diet-controlled. His past ocular history included a branch retinal vein occlusion in the right eye four years prior and a right sixth nerve palsy two years prior with spontaneous resolution of diplopia after 10 weeks. He did not smoke or consume alcohol. He worked as an automobile mechanic. On examination, BCVA was 20/20 in both eyes. The pupils were equal, round, and reactive to light without a relative afferent pupillary defect. IOP was 12 on the right and 14 on the left. Slit lamp examination was normal other than early nuclear sclerosis. Dilated posterior segment evaluation demonstrated pigment mottling of the superior macula on the right but was otherwise unremarkable. Ocular motility examination revealed a -2 abduction deficit on the right with an esotropia of 25 prism diopters in the right gaze, 10 prism diopters in the primary gaze, and 2 prism diopters in the left gaze. An MRI of the brain with and without contrast showed complete opacification and the enhancement of the right mastoid air cells and a portion of the middle ear cavity. The enhancing tissue in the mastoid air cells was abutting the right sigmoid sinus, likely representing bony erosion or dehiscence. Additionally, there was diffuse enhancement of the right tentorium; pachymeningeal enhancement overlying the right temporal, parietal, and occipital lobes; and leptomeningeal enhancement of the right cerebellum. A CT of the temporal bones also demonstrated an occlusive thrombus of the right transverse and sigmoid sinuses and jugular bulb. The patient was admitted to the hospital for the treatment of presumed infectious mastoiditis and received intravenous (IV) cefepime, vancomycin, and ampicillin/sulbactam. The following day, he was taken to the operating room by otolaryngology for a right mastoidectomy. Intraoperatively, no purulent material was found. The entire mastoid was filled with a soft tissue mass with a flesh coloration and consistency, which was removed and sent for culture and pathology. Antibiotics were discontinued two days later with no growth on culture (including testing for acid-fast bacilli). Pathology results from the biopsied mastoid tissue showed storiform fibrosis and a lymphoplasmacytic infiltration with an immunoglobulin G4 (IgG4) to IgG ratio of 48%. There was no evidence of lymphoma or plasma cell neoplasm by flow cytometry or immunohistochemistry. Serum levels of IgG4 subclass were elevated to 99.0 mg/dL (reference range: 4.0-86.0 mg/dL). He was discharged on a tapering dose of oral prednisone, and therapy with rituximab was initiated two weeks later. Six weeks after initial presentation, his ocular motility and alignment had returned to normal, and his double vision resolved completely.	
5. A 20-year-old female college student was referred to the neuro-ophthalmology clinic for the incidental discovery of bilateral optic nerve pallor on a routine eye examination. Three years prior to her presentation, she saw an optometrist for contact lens evaluation and was found to have mild pallor of both optic discs. Her vision was normal. She was referred to an outside hospital, where she underwent CT and MRI of the brain that were reportedly normal. One month prior to presentation, she saw an outside ophthalmologist for a routine eye examination, was noted to have visual field defects in both eyes, and was referred to the neuro-ophthalmology clinic. Her past ocular and medical history were unremarkable. She occasionally drinks alcohol, does not smoke, and does not take any medications. On examination, BCVA was 20/20 in each eye, and she had normal color vision and counted fingers in all quadrants with each eye. The pupils were equal, round, and reactive to light without relative afferent pupillary defect. IOP was normal in both eyes. External examination and anterior segment examination were normal. Dilated funduscopic examination showed mild temporal pallor of both optic discs, more prominent on the left. Humphrey visual field testing showed nonspecific peripheral defects infratemporally in the right eye and inferior arcuate visual field defect in the left eye.	Superior optic disc hypoplasia related to maternal type 1 diabetes
6. A 26-year-old female presented for the evaluation of transient visual obscurations. She reported constant poor quality of vision in both eyes for one month, with associated dimming and tunneling of her vision that occurred when turning her head in either direction or when quickly changing position. She also had constant, dull headaches, which were worse on lying down, and intermittent pulsatile tinnitus. She did not have diplopia. She had been in her normal state of health until about six months prior, when she developed pulsatile tinnitus and positional dizziness. Neuroimaging at that time demonstrated a large posterior fossa cyst that was associated with significant mass effect on her cerebellum and resultant Chiari malformation. Four months prior, she underwent fenestration of the cyst with neurosurgery. She did well in the immediate postoperative period and was discharged home on postoperative day 2. However, eight days later, she re-presented with wound drainage and underwent wound revision surgery. No cerebrospinal fluid (CSF) leak was identified, but cultures grew *Staphylococcus pseudintermedius*. Therefore, she was evaluated by the infectious disease team and was started on antibiotic treatment with vancomycin. A PICC line was placed, and she continued IV vancomycin treatment for six weeks. During that time, additional sensitivity analysis was performed, and the sensitivity of the organisms to minocycline was demonstrated. Therefore, her PICC line was removed, and oral treatment with minocycline was initiated, with a plan for at least six months of treatment. MRI completed two months postoperatively showed postoperative changes from the arachnoid cyst fenestration, with a small residual cystic fluid collection. On examination, visual acuity was 20/20 in the right eye and 20/20 in the left eye. There was no relative afferent pupillary defect. Extraocular motility was full. IOP was 23 mmHg in the right eye and 17 mmHg in the left eye. She correctly identified 13/13 Ishihara color plates with the right and left eyes. Anterior segment examination was unremarkable, and fundus examination demonstrated bilateral Frisen grade 4 optic disc swelling. Humphrey visual field 24-2 showed a few nasal missed spots in both eyes, with an enlarged blind spot in the left eye. The OCT of the retinal nerve fiber layer demonstrated thickening in both eyes, with average values of 338 microns in the right eye and 399 in the left eye. Her weight was 127 pounds, with body mass index of 21.	Pseudotumor cerebri secondary to minocycline
7. An 84-year-old male underwent uncomplicated cataract surgery in the right eye (RE) in late April. He then had cataract surgery on his left eye (LE) in early May. He noted difficulty reading at his one-week postoperative visit and was noted to have cystoid macular edema in the LE. He awoke in late June with slightly blurred vision in the LE and was noted to have optic disc edema in the LE at his one-month postoperative visit. His past medical history included gout, idiopathic cardiomyopathy, congestive heart failure, aortic regurgitation, aortic root dilatation, atrial fibrillation, hyperlipidemia, hypertension, prostate cancer, peripheral vascular disease, and erectile dysfunction. His medications were allopurinol, alendronate, amiodarone, atorvastatin, doxazosin, fluticasone, furosemide, metoprolol, and rivaroxaban. He denied symptoms of giant cell arteritis. He was a former smoker and drank about 1-2 alcoholic beverages several times a week. He was allergic to gabapentin, and his family history was noncontributory. Examination on July 1 showed that his visual acuities were 20/40 RE and 20/50 LE. He had a subtle left afferent pupillary defect. He identified 11/11 Ishihara plates RE and 10/11 LE. His examination demonstrated corneal verticillate, in both eyes, and his extraocular motility was normal. His cup-to-disc ratio was 0.05 RE and 0.0 LE. There was a normal optic disc RE and moderately severe disc edema LE, with no hemorrhages, lipid, or cotton wool spots. Visual field testing showed an arcuate visual field defect RE and a subtle central scotoma and inferior constriction LE. OCT showed a lamellar macular hole RE and significant macular edema LE, without vitreopapillary or vitreomacular traction in either eye.	U/L amiodarone-associated toxic optic neuropathy
8. A 74-year-old male was followed for two years by an ophthalmologist as a glaucoma suspect with asymmetric optic disc cupping (right greater than left). At the initial visit, visual acuities were 20/20 OD and 20/25 OS. IOPs were 17 mmHg OU. Automated static perimetry revealed scattered defects OD and superior and inferior arcuate scotomas OS. Repeat automated perimetry at eight months, 14 months, and 16.5 months later demonstrated possible progression of the visual field defects OS but with the appearance of the optic nerves, visual acuity, and IOP remaining stable. The patient's only visual complaint was a long-standing problem with glare and difficulty with driving at night. He did not particularly notice any problems with the vision in the left eye and denied any systemic or neurological symptoms. His medical history was notable for hypertension, hyperlipidemia, gout, osteoarthritis, nephrolithiasis, and multiple cutaneous basal cell carcinomas. He was taking aspirin, atenolol, lisinopril, atorvastatin, naproxen, and allopurinol. His ocular history was notable for early cataracts OU and a tonic pupil OD dating back to 12 years. He denied any family history of glaucoma or other eye disease.	Vitreomacular traction
9. A 62-year-old female presented for the evaluation of visual blurring from the right eye of two-week duration. She stated that her visual blurring got worse over the course of two weeks but since had plateaued. The left eye was unaffected. She denied diplopia, ptosis, facial numbness or paresthesias, jaw claudication, and scalp tenderness, as well as recent constitutional or systemic symptoms. Her past medical history was significant only for well-controlled hypertension and mild hyperlipidemia, for which she was on medications. There was no significant tobacco or alcohol use history. Ophthalmic examination revealed vision of 4/200 OD and 20/20 OS. Ishihara color plate testing showed no control plate OD and 14/14 plates OS. There was a right afferent pupillary defect. IOP was normal. External examination was normal without ptosis or proptosis. Extraocular motility showed mild limitation of ductions in all directions in the right eye and normal motility in the left eye. Alternate cover testing showed a small intermittent esotropia in the primary gaze. The rest of her cranial nerve examination was unremarkable. Slit lamp examination was remarkable only for mild nuclear sclerosis OU. Dilated fundus examination showed mild, diffuse optic disc swelling OD and normal disc OS with normal maculae, vessels, and periphery. Humphrey SITA Standard visual field with size V stimulus showed a dense central scotoma in the right eye and was normal for the left eye using the size III stimulus. OCT showed mild, diffuse retinal nerve fiber layer thickening OD and normal OS. Macular OCT was normal. Ganglion cell complex showed mild focal superior thinning OD and normal OS.	Idiopathic orbital inflammatory syndrome
10. A 47-year-old male presented for the evaluation of recent-onset diplopia, worse in the down gaze and lateral gaze. These symptoms were noticed one morning upon awakening two weeks prior to presentation. He described the images as being one on top of the other and slightly angled, and he felt his symptoms were stable since onset. He denied headaches, neck stiffness, associated pain, or blurred vision. The patient was in excellent health, and he had no past medical history of significance. Family and social histories were also unremarkable. Laboratory work ordered by his PCP, whom he saw initially, yielded a positive Lyme titer by western blot. The patient denied any history of tick bites, joint pain, or fevers; however, he did note recent fatigue. He also mentioned that as a child, he was told that he had a "wandering eye." He denied any prior patching or treatment for this. On examination, his visual acuity was 20/20 OU, with normal color and normally reactive pupils without an afferent pupillary defect. Visual fields by confrontation were full. He appeared to have a right head tilt. Ocular motility testing revealed full ocular ductions and versions. On prism alternate cover test, he had a 5Δ left hypertropia (LHT) in the primary gaze, which increased in the right gaze (8Δ LHT) and down gaze (6Δ LHT) and on left head tilt (6Δ LHT). There was no obvious excyclotorsion. He was able to fuse with 4Δ of base-down (BD) prism over the left eye.	Trochlear nerve palsy

All three large language AI models named OpenAI ChatGPT 3.5, Microsoft Bing, and Google Gemini received all 10 case descriptions directly as input, along with the following question: "What is the most probable diagnosis?" The public can easily access all three LLMs for free. Each question was asked separately for the ChatGPT 3.5, Microsoft Bing, and Google Gemini artificial intelligence programs, and a comparison was made between the responses and the neuro-ophthalmologist's actual diagnoses in the American Academy of Ophthalmology subspeciality program book.

To prevent any influence from earlier prompts and to eliminate memory bias, we have started a new chat for every prompt. For evaluation, the produced content was collected and organized. Next, we assessed how accurate ChatGPT 3.5, Bing, and Gemini were at diagnosing the problem. The first item was considered the most probable diagnosis because Google Gemini and Microsoft Bing frequently provided many differential diagnoses.

All chat box responses were completed in two separate categories: accuracy (correct or incorrect) and suitability (appropriate or inappropriate). All three chat box comments were graded as "appropriate" or "inappropriate," blind to the real information. A suitable description of the diagnosis differentiation procedure based on the input data in each case scenario was characterized as an "appropriate" response. Each response was further divided into "incorrect" and "correct" categories. Chat boxes that validated the diagnosis as being the same as the real fact were considered a "correct" response.

Since there were no human subjects in the study, it just used publicly available data and therefore did not need ethical approval.

## Results

Table 2 demonstrates the provisional diagnosis formulated by ChatGPT 3.5, Microsoft Bing, and Google Gemini for each case.

**Table 2:** Provisional diagnosis formulated by ChatGPT 3.5, Microsoft Bing, and Google Gemini for each case IgG, immunoglobulin G; d/t, due to

Case Number	Actual Diagnosis	ChatGPT 3.5 Diagnosis	Microsoft Bing Diagnosis	Google Gemini Diagnosis
1	Ethambutol optic neuropathy	Ethambutol-induced toxic neuropathy	Ethambutol-induced optic neuropathy	Ethambutol-induced optic neuropathy
2	Arteritic anterior ischemia optic neuropathy d/t giant cell arteritis	Giant cell arteritis (GCA) with associated anterior ischemic optic neuropathy (AION)	Giant cell arteritis (GCA), also known as temporal arteritis	Non-arteritic anterior ischemic optic neuropathy (NAION)
3	Optic neuritis in the setting of myelin oligodendrocyte glycoprotein (MOG) antibody-associated disease (MOGAD)	Acute optic neuritis	Acute optic neuritis	Acute optic neuritis
4	Sixth nerve palsy secondary to IgG4-related disease	IgG4-related disease involving the temporal bone specifically IgG-related mastoiditis	Petrous apicitis	IgG4-related disease
5	Superior optic disc hypoplasia related to maternal type 1 diabetes	Optic neuropathy	Optic atrophy	Nutritional optic neuropathy
6	Pseudotumor cerebri secondary to minocycline	Papilledema secondary to raised intracranial pressure (ICP)	Migraine	Idiopathic intracranial hypertension (IIH)
7	U/L amiodarone-associated toxic optic neuropathy	Postoperative cystoid macular edema (CME), along with optic disc edema and possibly amiodarone-induced corneal verticillate	Postoperative cystoid macular edema (CME) in the left eye, along with optic disc edema and possibly amiodarone-induced corneal verticillate	Postoperative cystoid macular edema (CME) along with optic disc edema and possibly amiodarone-induced corneal verticillate
8	Vitreomacular traction	Primary open-angle glaucoma (POAG)	Glaucoma	Optic neuritis
9	Idiopathic orbital inflammatory syndrome	Non-arteritic anterior ischemic optic neuropathy (NAION)	Non-arteritic anterior ischemic optic neuropathy (NAION)	Non-arteritic anterior ischemic optic neuropathy (NAION)
10	Trochlear nerve palsy	Fourth nerve (trochlear nerve) palsy	Fourth nerve (trochlear nerve) palsy	Trochlear nerve palsy

On the basis of the accuracy of diagnosis, all three chat boxes (ChatGPT 3.5, Microsoft Bing, and Google Gemini) gave the correct diagnosis in four (40%) out of 10 case scenarios, whereas in terms of suitability, ChatGPT 3.5, Microsoft Bing, and Google Gemini gave six (60%), five (50%), and five (50%) appropriate responses to 10 case scenarios, respectively. Table 3 represents the details of diagnoses in terms of both suitability and accuracy, which are provided by ChatGPT 3.5, Microsoft Bing, and Google Gemini.

**Table 3:** Responses from three chat boxes

Case Number	Response From ChatGPT 3.5		Response From Microsoft Bing		Response From Google Gemini	
	Suitability	Accuracy	Suitability	Accuracy	Suitability	Accuracy
1	Appropriate	Correct	Appropriate	Correct	Appropriate	Correct
2	Appropriate	Correct	Appropriate	Correct	Appropriate	Correct
3	Appropriate	Correct	Appropriate	Correct	Appropriate	Correct
4	Appropriate	Correct	Appropriate	Incorrect	Inappropriate	Correct
5	Inappropriate	Incorrect	Inappropriate	Incorrect	Inappropriate	Incorrect
6	Appropriate	Correct	Inappropriate	Incorrect	Appropriate	Correct
7	Inappropriate	Incorrect	Inappropriate	Incorrect	Inappropriate	Incorrect
8	Inappropriate	Incorrect	Inappropriate	Incorrect	Inappropriate	Incorrect
9	Inappropriate	Incorrect	Inappropriate	Incorrect	Inappropriate	Incorrect
10	Appropriate	Correct	Appropriate	Correct	Appropriate	Correct

The LLM chatbots' reaction length to the 10 chosen neuro-ophthalmology inquiries is mentioned in Table 4.

**Table 4:** The LLM chatbots' reaction length to the 10 chosen neuro-ophthalmology inquiries LLM: large language model

Response Length	ChatGPT 3.5 Mean ± SD	Microsoft Bing Mean ± SD	Google Gemini Mean ± SD
Word count	276.1 ± 37.76	272.6 ± 78.23	279.6 ± 73.89
Character count	1844.2 ± 305.58	1858 ± 491.97	2004.5 ± 510.31

## Discussion

Advances in artificial intelligence have had a big impact on a lot of different areas, including healthcare. Medical practitioners may be able to offer accurate and timely information with the use of conversational AI models. Because ChatGPT improves medical practitioners' whole clinical practice and keeps them informed of new advancements, it is a valuable resource for medical education. Research is still being conducted to determine how accurate ChatGPT is in applying the knowledge of medical specialties and subspecialties, despite the fact that it has advanced significantly since its founding.

Clinical knowledge is frequently predicated on current general expert consensus or standards, and treatment and monitoring are frequent components that change more quickly than disease description and pathogenesis. These features are challenging to include in a broad language model based on artificial intelligence since generative AI systems generally rely on pre-existing data for learning and subsequently use it to create new output.

Antaki et al. compared two versions of ChatGPT based on questions from Ophtho Questions and the Basic and Clinical Science Course Self-Assessment Program. The percentages of correctly answered questions for the prepared questions on a US board test were 49.2% and 59.4% [10].

Our findings show that the accuracy of all three conversation boxes is comparable. While ChatGPT outperforms Microsoft Bing and Google Gemini in terms of appropriateness or applicability, the results of our research do not align with the findings of earlier research carried out by Raimondi et al. [13] and Ali et al. [14], which demonstrated ChatGPT's superiority over alternative LLM options in examinations related to neurosurgery and ophthalmology, respectively.

Numerous distinctive features, such as ChatGPT's incredibly vast parameter set, the continuous input it gets from users and professionals to improve its training, its advanced reasoning and ability to follow instructions, and the use of more recent training data, are probably responsible for its superior performance in terms of appropriateness [15]. It is interesting to note, nevertheless, that in terms of accuracy, all three LLM chatbots were equally capable of providing perceptive responses.

Greater relevance, coherence, and quality in the model's outputs are made possible by its context breadth, which is the number of words it utilizes to build its response.

In our study, the average character count was lower in responses provided by ChatGPT in comparison to Microsoft Bing and Google Gemini.

Students seeking brief explanations may find that ChatGPT answers provide more concise feedback due to the shorter responses. Concise answers may also suggest increased computing efficiency in data processing, which would save time and resources [16].

There are a couple of inherent limitations with LLMs. First, there may be privacy issues raised when patient data is used for processing. Second, LLMs might yield inaccurate results because it was designed for public use rather than providing clinical diagnoses.

Furthermore, even if users repeatedly enter the same data into LLMs, it may still produce distinct results and primary diagnoses. This indicates that LLMs remain unreliable and are unable to offer consumers consistent recommendations and diagnoses [17].

Despite LLMs' incredible abilities in a multitude of domains, we must acknowledge its limitations.

Chatbots powered by artificial intelligence that are specifically programmed and trained to identify eye conditions are warranted. Additionally, it makes sense to deploy chatbots that can proactively request data that end users have not provided.

There are certain limitations to our study. On the basis of 10 case scenarios, we have assessed three LLMs. In order to further validate our results, other researchers might assess them using a larger number of cases. Furthermore, a number of these cases had typical disease presentations that could be definitively diagnosed. Thus, it is possible that this selection of cases does not accurately reflect neuro-ophthalmology practice, with many cases having complicating circumstances and questionable findings. On the other hand, a few examples showed unusual ways that the diagnosis was presented. This is when medical art enters the picture and calls for a human practitioner to carefully consider these aspects.

## Conclusions

The results demonstrate the great potential of artificial intelligence-driven chatbots, which can be used as a consultation tool to help family doctors and patients get referral recommendations. For preliminary evaluations in tertiary ophthalmology care, these models can also be helpful. Additionally, students studying neuro-ophthalmology may find AI models to be an additional instructional resource that enhances standard learning environments by offering real-time, data-driven feedback.

The growing importance of AI in medicine makes it important to keep in mind the moral and professional responsibilities that doctors have. Technology needs to be utilized in conjunction with experts, not as a substitute for them.
